# How Large Is the Metabolome? A Critical Analysis of Data Exchange Practices in Chemistry

**DOI:** 10.1371/journal.pone.0005440

**Published:** 2009-05-05

**Authors:** Tobias Kind, Martin Scholz, Oliver Fiehn

**Affiliations:** University of California Davis, Genome Center – Metabolomics, Davis, California, United States of America; Texas A&M University, United States of America

## Abstract

**Background:**

Calculating the metabolome size of species by genome-guided reconstruction of metabolic pathways misses all products from orphan genes and from enzymes lacking annotated genes. Hence, metabolomes need to be determined experimentally. Annotations by mass spectrometry would greatly benefit if peer-reviewed public databases could be queried to compile target lists of structures that already have been reported for a given species. We detail current obstacles to compile such a knowledge base of metabolites.

**Results:**

As an example, results are presented for rice. Two rice (*oryza sativa*) subspecies have been fully sequenced, *oryza japonica* and *oryza indica*. Several major small molecule databases were compared for listing known rice metabolites comprising PubChem, Chemical Abstracts, Beilstein, Patent databases, Dictionary of Natural Products, SetupX/BinBase, KNApSAcK DB, and finally those databases which were obtained by computational approaches, i.e. RiceCyc, KEGG, and Reactome. More than 5,000 small molecules were retrieved when searching these databases. Unfortunately, most often, genuine rice metabolites were retrieved together with non-metabolite database entries such as pesticides. Overlaps from database compound lists were very difficult to compare because structures were either not encoded in machine-readable format or because compound identifiers were not cross-referenced between databases.

**Conclusions:**

We conclude that present databases are not capable of comprehensively retrieving all known metabolites. Metabolome lists are yet mostly restricted to genome-reconstructed pathways. We suggest that providers of (bio)chemical databases enrich their database identifiers to PubChem IDs and InChIKeys to enable cross-database queries. In addition, peer-reviewed journal repositories need to mandate submission of structures and spectra in machine readable format to allow automated semantic annotation of articles containing chemical structures. Such changes in publication standards and database architectures will enable researchers to compile current knowledge about the metabolome of species, which may extend to derived information such as spectral libraries, organ-specific metabolites, and cross-study comparisons.

## Introduction

Unlike animals, plants produce all metabolites by their cellular biochemical machinery from carbon dioxide and inorganic nutrients. Hence, well-studied plant species should present a best-case scenario for retrieving a comprehensive overview about all metabolites that have previously been reported and identified, or that can be deduced to be present by pathway reconstruction from genomic information. We have thus chosen rice (*oryza sativa*) with its two subspecies *oryza japonica* and *oryza indica* as test case.

**Figure 1:**
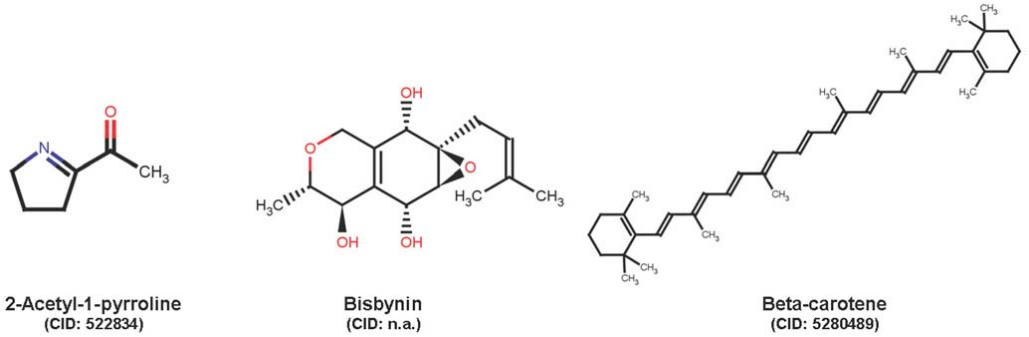
Metabolites found in *oryza sativa* plant organs. The flavor of basmati rice comes from 2-acetyl-1-pyrrolidine, beta-carotene enriched golden rice was created to defeat vitamin-A deficiencies in the third world and the compound Bisbynin is not created by rice itself but a parasitic soil fungus *Stachybotrys bisbyi* found in rice seeds.

Due to the importance of rice as one of the world's staple foods [1], the question arises: what is naturally found in rice (see Figure 1)? How many primary and secondary metabolites can be found in *oryza sativa* species that are established in the literature and databases? What kind of structural and spectral information for rice compounds has already been collected? Are *de novo* metabolite identifications needed for every species that is subjected to metabolome studies?

**Figure 2:**
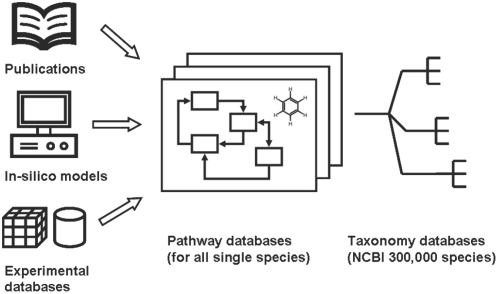
The process of building pathway and metabolite databases includes a) data extraction from the literature b) use of in-silico and ortholog mapping approaches and c) direct input from experimental databases like SetupX. Molecular pathway databases can be built for all known taxonomic species which can be found in the NCBI taxonomy database.

For genome sequenced species, information about taxonomy specific metabolic pathways and metabolites can be compiled in pathway databases. Figure 2 shows that there are three general approaches: information retrieved from literature, from genome annotations or from experimental data. The first method obtains pathways and chemical structures from the peer-reviewed scientific literature by manual or automatic curation. A second way is to use in-silico methods and methods similar to gene ortholog mapping approaches and deducing metabolites using computational methods. The third approach is to obtain metabolite data directly from experimental result databases together with a complete study design and metadata. For this experimental approach, it is critical to unambiguously identify the chemical structures of the detected metabolites as these compounds will then be used to assign enzyme E.C. numbers and subsequently be used to annotate gene functions. It is therefore crucial to include target lists of known compounds in order to avoid redundant rediscovery. Preferentially, molecule target lists would be further constrained by information of taxonomy and organs. Structure elucidation of unknown small molecules is a tedious and complex process requiring sophisticated chromatographic separations [2] with multiple structural assignment tools such as mass spectrometry (MS) and nuclear magnetic resonance (NMR). Spectral information and other experimental physicochemical descriptors need to be collected and disseminated for each single compound.

We recently introduced approaches to use target molecule databases [3] for metabolic profiling purposes and annotation of unknown compounds [4]. To perform such annotations the correct molecular structure and if possible mass spectra must be obtained from database queries. From the molecular structure the accurate masses and isotopic abundances can be used and searched in experimental data sets. Additionally substance data can be matched against in-silico generated tandem mass spectra [5] or can be used in approaches using substructure detection algorithms and molecular isomer generators [6]. In order to limit the search space of compounds, such database approaches would benefit if information was given in which organisms (coded as taxonomy source) and in which organs or cell types these metabolites were known to exist.

We here present results of a query of several databases combining all three approaches to metabolome compilation (literature, in-silico pathways and experimental data), using *oryza sativa* as example. We explain the difficulties in this process and discuss solutions to overcome the current procrastination and stagnation of data exchange practices in chemistry.

## Methods

Searches were performed in October 2008. Results may not always reflect database updates that were released after this date. Due to the complexity of steps in cleaning and curating result data sets, actual numbers may differ to some degree if queries are repeated, while the overall conclusion on limitations with current database designs will be found reproducible.

The CAS Chemical Abstracts Database [7] search was performed with SciFinder 2006 and 2007 version for Windows. Chemical Abstracts covers most of the chemical and patent literature since 1907. Annual institutional subscription is required for this database. Results were exported as CAS numbers and text. Analysis of substances count was performed within SciFinder. Search term was ‘oryza sativa’. To further constrain the search, ‘liquid chromatography’, ‘gas chromatography’, and ‘mass spectrometry’ were used as combined search terms using the intelligent query language (“Oryza sativa and GC-MS” not pesticides”). Histograms and categorizations and patent searches were performed with SciFinder.

The CRC Press Dictionary of Natural Products search was performed using the web frontend (DNP 17.1 Copyright 2008 Taylor & Francis Group) [8]. Annual institutional subscription is required for this database. Search terms for biological source: ‘oryzae’ and ‘*oryza sativa*’.

The PubChem database was searched with the terms ‘*oryza sativa*’ in the compound and substance section. Furthermore it was accessed via the National Center for Biotechnology Information (NCBI) Entrez search portal [9]. As the direct search resulted in zero hits (no taxonomy data is stored in PubChem) the search was performed via PubMed and PubMed Central [10]. PubMed Central was searched either with the term ‘*oryza sativa*’ or the taxonomy ID ‘txid4530’ [Organism:exp]. Substances in PubChem were shown with the display substance option in PubMed.

The Beilstein database (Elsevier Information Systems GmbH) [11] was searched with the MDL CrossFire Commander 7.0 frontend. Annual institutional subscription is required for this database. The search term ‘*oryza sativa*’ was used.

Dr. Duke's Phytochemical and Ethnobotanical Database [12] is a free factual database hosted by the Agricultural Research Service (ARS) at the U.S. Department of Agriculture (USDA). The latest update of the database was 1996. The search term ‘*oryza sativa*’ was used.

SetupX [13] is a study design database for metabolomic projects. It currently contains raw data and assigned meta-data from GCTOF mass spectrometry metabolic profiling experiments that were annotated by the BinBase mass spectral database [14]. Currently 40 species are covered and more than 14,000 public and non-public experimental datasets are available. The search term ‘*oryza sativa*’ was used.

The RiceCyc database [15] is developed and curated by Gramene (Resource for Comparative Grass Genomics) [16]. RiceCyc is a catalog of known and predicted biochemical pathways from rice (*oryza sativa*). The database (version 2.0.2; *oryza sativa japonica*; strain: nipponbare) was fully downloaded and data was analyzed as text.

The KEGG database [17] is an important pathway and metabolic network database covering a wide range of organisms. The database was queried with the available online tools and content was downloaded in raw format and processed for species ‘*oryza sativa*’, coded as *osa* and *eosa* string.

The KNApSAcK database [18] is a species-metabolite relationship database containing 23,287 metabolite entries and 46,093 metabolite-species pair entries (version 1.200.02). It was searched via the web frontend [19]. Mostly secondary plant metabolites are covered.

The Reactome database [20] (version 26) was queried using ‘*oryza sativa*’ as search term. Reactome contains curated human pathways and additional 22 non-human species with parts of the data obtained by orthology mapping approaches for genes and proteins in case whole-genome sequence data was available.

For patent searches the CAS database [7], the IBM Chemical Search alpha [21] and IBM Chemical Patent search beta and the SureChem patent database [22] were used. The CAS DB and SureChem DB allowed factual searches with the keywords ‘*oryza sativa*’ or ‘rice’ and also Markush structure search [23] which is specifically used for patent searches. Surechem allows subscription based download of structures found in patents. In our case the IBM patent database was used to export all detected chemical structures from all world patents (WIPO).

## Results

We here report the results for each database query and discuss search capabilities and problems (Table 1). Mostly organic molecules are reported, leaving out the majority of metals and other rare earth elements. Data on metals and trace metals can be relatively easily acquired with inductively coupled plasma mass spectrometry (ICP-MS) which is not the case for small organic molecules. Molecules larger than 2000 Dalton and special compound classes like glycans and peptides are also excluded. The analysis of those substance classes are covered by glycomics and proteomics based techniques. All retrieved results with 112,000 factual data points on rice metabolites reported here can be downloaded from the supplement section at the authors' homepage [24].

**Table 1:** Number of small molecules found in *oryza sativa* by database search.

	Database	Oryzae compounds	Mix-up	Comment
**Compound DBs**				
	PubChem/PubMed	>3000	mixed	contains pesticides and drugs
	CAS	>1400	mixed	contains pesticides and drugs
	Beilstein	554	mixed	contains pesticides and drugs
	SetupX	268	clean	with relative concentrations
	Dr. Duke	201	clean	with relative concentrations
	DNP	55	clean	few mixed with multi species
	KNApSAcK DB	48	clean	few other species included
**Ortholog/Pathway DBs**				
	KEGG	3661	mixed	putative compounds included
	RiceCyc	1500	mixed	putative compounds included
	Reactome	396	mixed	putative compounds included
**Patent DBs**	IBM Patent Search	9780	mixed	contains pesticides and drugs

The table reports all organic metabolites with possible organism mix-ups including bacterial and fungal metabolites or pesticides and drugs. Search date October 2008.

### Dictionary of Natural Products

The commercially available Dictionary of Natural Products was used over its online web frontend [8]. The web frontend allows structure and substructure searches as well as text searches. The DNP database itself covers most of the natural product publications and small molecules and peptide structures from animals, plants and fungi. DNP is the largest curated database of natural products covering more than 200,000 entries. Taxonomic names and physicochemical properties and secondary literature are included for most of the structures. No connections or links to other life sciences databases exists. One major caveat is that chemical structures cannot be downloaded in bulk but only one-by-one. Moreover, structures are only shown as graphics and not as chemical structure files which would be necessary to computationally analyze and compare compounds. However, only 55 metabolites were retrieved when querying DNP for metabolites in rice using the DNP taxonomy search. This number is certainly too low as, for example, many proteinogenic amino acids and other primary metabolites are not included in DNP which focuses on secondary metabolism. Common metabolites like arginine are covered in DNP and can be found in rice, but no taxonomy information is assigned. The overview results also contained chemicals which live either in symbiosis or as parasites. An additional fulltext search obtained 224 matches with links to possible parasites and bacteria related to rice. Only by evaluating each single source it is possible to filter those out. An example would be the Bisbynin [25] which is a metabolite of the fungus *Stachybotrys bisbyi* from seeds of *Oryza sativa* (see Figure 1).

### Chemical Abstracts Database and SciFinder

The Chemical Abstracts Database is the largest curated database of chemical and biomedical literature [7]. As of October 2008 it contains 39 million organic and inorganic substances, 60 million protein sequences and additionally paper abstracts. The major difference to open abstracts databases like PubMed is that not only abstracts are covered but all chemical and biochemical literature is read in full text by computer algorithms to detect chemicals structures in text and pictures. Trained chemists further curate those structures. The CAS database has no direct taxonomic annotation but has a specialized index database which also includes taxonomy names. More than unique 42,115 literature abstracts were obtained by searching for *oryza sativa*, but only 5,000 records at one time can be used and stored for obtaining chemical structures by the license agreement. Additionally, these results must be deleted after the research project is finished. These constraints limit the use of SciFinder for metabolic profiling approaches despite the good data quality. Moreover, chemical structures can only be manually downloaded one-by-one. No list matching (batch approach) for structure search is possible. The number of structures obtained from CAS queries is depending on the exact search terms and refinements. For example, constraining the search using “golden rice” as search term resulted in only twenty-seven chemicals, among them beta-carotene, trans-luteine, cholesterol and other terpenoids. Importantly, metabolite structures are mixed up with pesticides like Malathion and Chlorpyrifos. Refinement of such lists is very difficult as cross-referencing CAS hits and links to compound identifiers in other databases (such as KEGG, PubChem) are not given. It also must be mentioned that although the CAS DB has the largest coverage of substances from the chemical and bio-chemical literature it is by no means complete [26]. In many instances the original publication contained multiple structures which could not be found in the database. The SciFinder 2007 version has a much finer grained and useful categorization tool however that does not solve the problem of results mixed with drugs, protein sequences and pesticides. Furthermore CAS numbers may change without notice. For example, the rice metabolite Ribosylnicotinamide had been assigned with more than one CAS number, i.e. 19131-72-7, 20299-13-2, 954368-04-8 and 1341-23-7. Many outdated CAS numbers can be still found through internet or DB queries even they are no longer supported by CAS itself.

### PubChem

PubChem is the largest open access database for small molecules. It is part of a set of life sciences databases hosted at the US National Center for Biotechnology Information [27]. As of October 2008 PubChem contained 47 million substance records and 19 million unique compound records. It is regarded as a premier database for small molecule research. Structures can be freely downloaded and links to relevant databases are provided. One major drawback is that PubChem and PubMed lack chemical information from full text chemical publications as provided in the Chemical Abstracts Database. A general overview about pointers to gene sequence, protein and small molecule resources can be obtained at NCBI Entrez which also links to PubChem and many other NCBI databases. Entrez lists 1,232,016 expressed sequence tag records (EST), 355,026 Genome Survey Sequence records (GSS) and 231,927 records in protein sequence databases for rice but no chemical substances in the PubChem record. Furthermore taxonomy-related queries cannot directly be submitted to PubChem. Instead, PubMed and PubMed Central need to be searched for taxonomy data, and subsequently, related substance records need to be displayed. Unfortunately, the results present all substances retrieved from the research papers but do not detail whether or not the compounds occur as genuine metabolites of the queried organism. For example the hit ‘Omeprazol’ is clearly not a rice metabolite as it refers a synthetic drug for acid reflux treatment. Furthermore the 11,490 articles in PubMed and the 4,472 open access article in PubMed Central reflect only one third of the chemical literature covering rice research as retrieved from CAS searches.

### Beilstein database

The Beilstein database covers 10 million compounds and organic reactions and contains 500 million experimental chemical property values (factual information). Export of chemical structures as chemical structure file (SDF) file is possible. Only one hundred records can be exported at a time. 554 substance records were retrieved from querying the Beilstein DB with ‘oryza sativa’ but results were mixed with hits on pesticides and growth inhibitors.

### Dr. Duke's

Dr. Duke's Phytochemical and Ethnobotanical Database contains 201 organic and inorganic chemicals found specifically only in rice. Structures are presented as names only. Taxonomy searches are possible for a variety of plants. Concentrations for chemicals in different compartments (fruit, hay, juice, leaf, petiole, plant, protoplast, root, seed, shoot, sprout seedling, stem, tissue culture, wax) are given. Concentrations are recorded in parts per million (ppm). No external links or cross-references to other databases and other compound identifiers exist and the database is not updated anymore.

### SetupX

SetupX/BinBase comprises 268 unique metabolites for oryza sativa. The data was obtained from the non-public part of the database from GC-MS metabolic profiling experiments. The datasets contain 108 primary metabolites with relative concentrations which were identified by authentic chemical standards. Additionally around 160 unique unknown metabolites could be assigned with help of the BinBase algorithm via mass spectral and retention index matching. Raw and processed experimental datasets can be downloaded concomitant with a complete experimental description and report. Exploring phylogenetic relationships in SetupX/BinBase is facilitated as the taxonomic structure is based on NCBI taxonomy database [29] while allowing manual curation of entries. Hence, SetupX/BinBase database requests enable querying which unique substances were experimentally determined in two different subspecies and which metabolites were fully unique in a specific taxonomy kingdom. Apart from NCBI, SetupX uses further plant [30] and animal ontologies [31]. All hits with identified structures are referenced to PubChem and KEGG. However, few secondary metabolites are included, and the abundance of metabolites without elucidated chemical structures limits use of SetupX/BinBase for further biochemical or physiological interpretations.

### RiceCyc

RiceCyc is a new compound and pathway database obtained from known literature pathways and by computational approaches obtained from the fully sequenced rice genomes. Datasets were obtained using the Stanford Research Institute SRI PathWay tools [32] and by later curating the database with Arabidopsis-rice ortholog annotations. The database statistics shows 1,500 annotated compounds with 1,463 compounds encoded in SMILES structure format [33]. Although SMILES formats are not as universal as the newer International Chemical Identifier codes (InChI) [34], but at least SMILES structures are machine readable and thus can in principle be compared to hit lists from other databases. Unfortunately RiceCyc has no direct link-out to PubChem and only 580 of those structures have assigned CAS numbers, so direct overlap analysis of metabolites retrieved from RiceCyc to compounds from other databases are very difficult at this point. Nevertheless, RiceCyc is certainly a very valuable tool on retrieving information which metabolites can be expected in rice samples, although not yet supported through experimental or literature evidence.

### KEGG

KEGG is a well known metabolite and pathway database. The current version covers 91,879 pathways generated from 240 reference pathways and around 850 taxonomic species with 10,303 reactant pairs and 15,217 metabolites. Recently, coverage of lipid metabolism was improved with inclusion and links to LipidMaps databases [35] and missing secondary plant metabolites are now obtained from the KNApSAcK DB. Furthermore KEGG is indexed in PubChem and provides multiple outlinks to other databases. With KEGG Brite [36], a collection of hierarchical classifications representing the knowledge on various aspects of biological systems, the database possesses a powerful query frontend. Altogether 3,661 compounds were obtained for rice based on pathway mappings. As with all in-silico approaches there is no guarantee that computed substances truly occur in the species. For example, KEGG predicts the bacterial metabolite Rhodopinal or the chemotherapeutic anti-cancer drug Tegafur to be found in rice, which appears unlikely to refer to genuine plant metabolites without experimental proof. Nevertheless, plants are able to metabolize xenobiotic drugs and pesticides; therefore, the inclusion of such compounds in pathway databases is important to obtain a complete picture of metabolism. An important curation of KEGG and other metabolic repositories would be to distinguish between authentic organism-specific metabolites and xenobiotic compounds.

### KNApSAcK DB

The KNApSAcK species-metabolite database search resulted in 48 compounds, most of them referring to secondary metabolism. The database contains no outlinks to other databases but the KEGG database links some of its results to the KNApSAcK DB. The database contains a simple text search and web search frontend. Molecules are coded as pictures and in some cases contain also CAS numbers. Around 600 taxonomic species are currently covered with 30 species having more than one hundred metabolites assigned, whereas the median of the number of assigned metabolites is four.

### Reactome DB

The Reactome pathway database covered 2,836 proteins, 865 reactions and 447 pathways and 396 metabolites for rice. Reactome allows the download in SBML and BioPax format as well as ChEBI and KEGG identifiers for single reaction maps but also for specific taxonomic species.

### Patent databases

A patent search in the CAS database revealed around 3,000 patents dealing with rice. The SureChem DB revealed 2,531 US patent office granted patents, 609 European granted patents and 2,385 world patents. The IBM Chemical patent search was used to export chemical structures from 1,384 covered world patents issued by the World Intellectual Property Organization. A total of 69,215 chemical compounds were mentioned and 9,780 unique chemicals were obtained after refinement with Instant-JChem [37] using chemical structure overlap analysis. Patents as sources for extracting molecular biological knowledge are unreliable because of the different scope of patent databases [38]. Usually they also contain molecules from secondary literature with no proof if such molecules were indeed found in rice. Without manual curation it is not possible to separate between genuine rice compounds and herbicides and pesticides and other chemicals mentioned in patents.

### Additional metabolite databases

MetaCyc and BioCyc [39] are umbrella databases covering 1,500 organisms and 1,100 metabolic pathways and the collection of Pathway/Genome Databases. These databases were not used for searches, because the *oryza sativa* metabolite and pathway information are explicitly stored in the RiceCyc database. The integrated rice science database Oryzabase [40] does not contain small molecule information but mostly gene annotations. The MetaCrop [41] database contains information about rice obtained from the literature and multiple other pathway databases. The whole database contains around 300 metabolites but most of the other information is enzyme related and it is not possible to refine metabolites which only occur in rice. A very important feature of the MetaCrop database is that for some substances and enzymes it contains data on developmental state of plant, organs, tissue and compartment together with a related link to the literature on PubMed. The National Library of Medicine Medical Subject Headings (MeSH) contains only a small subset of annotated organisms. No information is included if a certain metabolite is genuine and specifically found in single species. Other metabolite databases like the Madison Metabolomics Consortium Database [42], the Human Metabolome Database [43] or the Metlin database [44] and ChEBI [45] were not included because they contained no *oryza sativa* taxonomy information or resulted in zero hits.

## Discussion

It was not possible to automatically compute a single combined large knowledge repository of all small molecules of rice (*oryza sativa*) which would comprise both experimentally verified compounds and putative compounds annotated from genomic and pathway databases. Three major obstacles were hindering this approach:

1. A number of databases did not allow or did not enable batch downloads and storage of compound lists and structures.
2. A number of databases did not cross-reference compound identifiers or did not export structures in machine-readable formats, so that analysis of overlaps of hit lists was not possible.
3. Many databases did not distinguish between metabolites that are produced by the biochemical machinery in rice from xenobiotic molecules that were either found in collateral experiment designs in literature or as putative compounds in pathway databases.

We first suggest a paradigm change in publishing metabolite information and subsequently explain how such change would alleviate the problems listed above.

### Small molecule reports need to be annotated by PubChem CIDs and structures

Where extensive data on small molecules are presented in chemical reports, structures should be annotated according to compound identifiers in the authoritative database PubChem. In case structures are not yet included in PubChem, these should be encoded in a chemical structure to allow database search requests and chemical overlap calculations. The best way to represent a chemical compound is not by a name or even a database identifier, but by its structure encoded in Structure Data Format (SDF MDL V2000) or the open Chemical Markup Language (CML) format or InChI codes. A few databases already provide the IUPAC/NIST standard of InChI codes [50] or the shorter hashed InChIKey [51]. The new InChIKey resolver services [52] implemented by the Royal Society of Chemistry (RSC) [53] and Chemspider [28] allows to create InChIKeys from molecular structures and a reverse lookup of InChIKeys to obtain the associated known structures from molecular databases. The InChIKey can be used for web based literature search and also for chemical database search and merging of compound lists from multiple sources [54]. Some other databases support the SMILES code for structures. The use of SMILES code is not recommended because multiple vendors create different representations of the SMILES code. Also true canonical (unique) SMILES are vendor specific. The machine-readable structure hit lists could in principle be used to obtain a library containing chemical connectivity information and spectral meta-data. The Metabolomics Standards Initiative (MSI) suggests reporting metabolites using unique chemical identifiers like InChI Codes or PubChem compound IDs [55]. Both the MSI and the broader community initiative MIBBI (Minimum Information for Biological and Biomedical Investigations) [56], [57] lack the power to enforce requirements to report and publish metabolite data and metadata (e.g. NMR and MS spectra) in electronic formats but are good-will projects with recommendations and lead-by-example standards. The same can be said about the Blue Obelisk movement which supports interoperability in chemical informatics by promoting reusable chemistry via open source, open data, and open standards [58]. In addition, spectral data could be added to PubChem identifiers using the open XML spectral exchange format CMLSpect [72] or open source tools (project SPECTRa) [73]. However, at this point, spectral data depositions in open exchange formats cannot be made mandatory, as some of the open exchange formats cannot handle complex multistage chromatographic data sets, such as LC-MS with MS^2^ and MS^3^ scans and ion trees. In such a case the submission of the raw files in vendor specific formats seems to be sufficient.

### Small molecule reports need to be public and machine-readable

**Figure 3:**
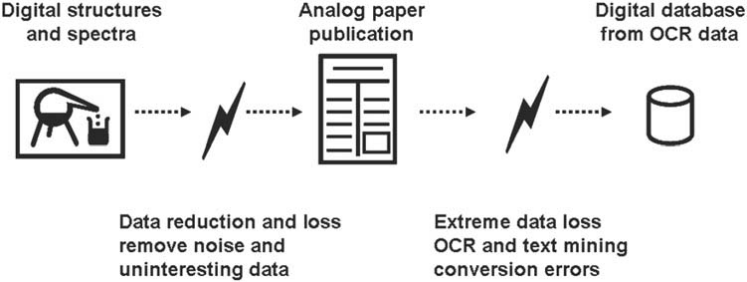
Data loss occurs during conversion of digital data in the lab to analog data in a publication. Later analog information from a publication including structures and molecular spectra are converted back to digital information (hamburger-to-cow algorithm). Such name to structure and OCR-optical chemical structure recognition algorithms are error prone and information loss is even higher for complex molecular spectra. Direct electronic submission of chemical structures and spectral data is recommended.

Chemical structures and spectra should be made available in machine readable formats and provided by either the submitting authors or the publisher. Journals should ensure data submission before accepting manuscripts and funding agencies should require submission of metabolite metadata in their funding guidelines. The Royal Society of Chemistry (RSC) with Project Prospect [74] and two *Nature* journals have shown that substance and ontology annotations can be done successfully. The current approaches to compile chemical knowledge repositories use manually and automated full-text curation and optical character recognition to obtain structures and spectra from graphics and text, as in the Beilstein Handbook of Chemistry from 1881 and the Chemical Abstracts Service starting in 1907 [61], [62]. The current approach is shown in Figure 3. Experimental data are digitally recorded in a laboratory and condensed into a paper publication. During that process, attention is focused to the main point of a scientific paper, mostly by excluding data and reducing noise. A major problem is that data is also removed which might be interesting for other researchers in a different context, e.g. metabolic concentrations that do not change after a perturbation. Results are published on paper or electronic (bitmap PDF) documents, hence transforming digital into analog data. If metabolite structures and spectra are converted back from analog into digital form, optical structure recognition and text to structure algorithms are used which are very error prone. In some cases authors are already required to submit machine readable structures and spectral data directly to the supplement section of a journal. In such a case publishers should not transform machine readable data back to bitmap PDF and destroy the semantic content. That is especially important for data tables, chemical reaction drawings and high resolution spectra in supplemental data. Additionally to the provided semantic presentation of a given journal article the PDF format can serve as a medium to distribute compact, platform-independent documents that are easy to read and print.

Even today, primary documents as PDF and Microsoft Word files can store XML (metadata) as part of the document structure [63], and molecular drawing software programs such as ChemWriter, GChemPaint and ACD ChemSketch can attach the molecular connection table to the graphics [64]. Next-generation MS Word implementations will encode chemical structures and more important chemical reaction data directly in the MS Word document format (Chem4Word) [65]. In this way it will be possible to store and expose chemical information in a semantically rich manner and support data mining scenarios for authors, readers and publishers. Hence every chemistry publication will be automatically structure searchable. The semantic annotation tool Oscar3 could then be used to annotate publications in high-throughput mode with additional XML data and add semantics and ontology support [66]. Client side applications for web browsers will be used which enrich published chemistry texts using resource description frameworks (RDF) and web ontology languages [67] and would profit from additional metadata. For past publications, high quality OCR techniques will still be needed using chemical structure recognition algorithms like OSRA [68], Kekule [69], CLiDE [70], ChemOCR [54] and ChemReader [71] as well as additional name-to-structure and structure-to-name approaches. However, the current approach of parsing peer-reviewed literature to obtain structures and important spectral data sets from bitmap data is not keeping up with the today's technological possibilities.

### Small molecule reports need to disclose sample metadata and absolute concentrations

Metabolite concentrations should be annotated with correct taxonomical and biological metadata, e.g. for the organs, cells, liquids or subcellular compartments that were studied. Stricter rules for publications have long been established in genomic research which requires submission to standard repositories to GenBank [60] and submission of gene expression or microarray data to the Gene Expression Omnibus (GEO) database. That is not the case in chemistry, biochemistry and metabolomics. As the NCBI taxonomy database [29] is linked to PubChem, each of the 300,000 unique taxonomic species in NCBI could potentially be annotated with PubChem data and fulltext PubMed Central data if this is financially, legally and administratively supported by NIH. An alternative solution would require that journals perform annotation of compounds and annotation within PubChem automatically. Working solutions are already implemented in some of the Nature journals (*Nature Chemical Biology* and *Nature Chemistry*) which submits all chemical structures to PubChem and in the RSC Project Prospect which performs online annotations of chemicals and ontology data [59] and substances with their articles DOI links can be searched via ChemSpider. With such metadata and additional information on absolute (molar) concentrations, researchers could query reports for cross-species, cross-organ and cross-study comparisons. When relative metabolite changes are reported, comparisons between different analytical platforms are not feasible unless a unified reference sample would be used.

### Benefits of authoritative data repositories

How would the implementation of these publication standards benefit researchers and remove the obstacles to retrieve the current knowledge on a species metabolome? Batch downloads from repositories will immediately become easier and richer, once small molecule reports are machine readable with PubChem CIDs and chemical structures. Chemistry search engines like ChemSpider will collate information and make it widely available, similar to current public databases like MetaCyc and PubChem. Unfortunately, many database suppliers will continue to have financial or legal restrictions that prevent large public downloads. Even if open access may eventually decrease the importance of proprietary databases, funding and curating open access databases will remain a problem, potentially resulting in a difference between public and privileged database access. Therefore, publications with electronic metadata and structures can only be the first step to improve data accessibility. We are convinced that such strategies will change the structure of existing databases in a very short time frame. For example, BioCyc has recently added PubChem identifiers and also KEGG is fully linked, but had not supported PubChem initially. Ultimatively authoritative data repositories are needed just like GenBank or the Gene Expression Omnibus (GEO) database.

### Differentiation between endogenous and exogenous metabolites

The differentiation between endogenous and exogenous metabolites is far more difficult. In-silico approaches based on orthology mapping or computational approaches, i.e. the RiceCyc and KEGG databases, contained the largest computationally compiled for rice metabolites, many of which are supposed to occur in samples. Until recently, lipids were mostly absent from pathway databases but are now included due to the new LipidMaps repository [35]. KEGG contained most of the polar and non polar lipids with general coverage of around 1,500 lipids mostly obtained from LipidMaps. It is important to test experimentally (using analytical chemistry tools) whether these metabolites and pathways are actually correctly predicted by pathway databases. Tools exist like the Pathway Tools Omics-Viewer [47] which allow the mapping of experimental data of identified metabolites onto pathways. Conversely, once novel metabolites are unambiguously identified in species like rice, it is important that genome databases provide tools to suggest potential enzymes and genes that could link these novel metabolites to known pathways via a minimum set of chemical transformations. Such efforts would only be possible if structure information is encoded in InChI formats and supported by genome database providers. Related efforts have been reported on ‘atomic reconstruction maps’ which are constructed on a substructure level [48] and carbon-fate maps for metabolic reactions [49].

Only four experimental data repositories resulted in hits excluding xenobiotic compounds, SetupX/BinBase, Dr. Duke, DNP and KNApSAcK DB. However, the number of retrieved metabolites was very small for these databases and thus very likely cover only a small fraction of the real size of the rice metabolome. Conversely, databases like CAS, PubChem and Beilstein contained a large number of metabolites, but results were mixed with drugs or pesticides and thus also do not allow compiling an overview of genuine rice metabolites. Unfortunately, our suggestions for changes in publication standards will not solve the relationship of ‘chemicals’ in the reports to the specific organisms, as this would require some form of ontology or hierarchy in the database entries as well as in journal reports. While in plants, most (but not all) experimentally detected small molecules can be assumed to be synthesized by the plant biochemical machinery, such assumption is certainly not true in humans. For animals, the distinction between exogenous and endogenous metabolites is far more arbitrary and also less meaningful. Xenobiotic compounds can be metabolized by plants and such pathways would be important for herbicide design and in phytoremediation processes when plants are used for soil clean-up. Common cheminformatics tasks during database curation like multiple stereoisomers, different salt forms and tautomer normalization [46] and compound overlap calculations were not investigated in this report due to the multiple problems in the first place.

### A paradigm change is inevitable and possible

A paradigm change for publishing chemicals structures, spectra and molecular properties is inevitable. Implementing new paradigms or new standards in databases and journals often seems too difficult to achieve. Nevertheless, there are multiple success stories in biological sciences, from the protein databank (PDB) to GenBank and the Gene Expression Omnibus to crystal structures. Although use of such standards seem to be of little benefit for the individual researcher at first glance, once public databases can be queried across studies, benefits will be obvious even for small datasets, e.g. to prove the novelty of findings for a specific (small-scale) experiment.

### Conclusions

In order to obtain an open access and free full coverage of all rice metabolites, approximately 50,000 journal articles would be needed to be reanalyzed using fulltext chemical annotation services. Once such a database would be established, further updates on novel identified metabolites should use a radically different approach. While we have taken *oryza sativa* as an example with the RiceCyc database already in place, this call for a paradigm change extends to all 300,000 species currently comprised in the NCBI taxonomy database. While one option to improve the situation would be to compile databases from literature data (i.e. bitmap PDF documents) using error-prone optical character recognition (OCR), free access databases such as ChemSpider and PubChem would be better suited to store compound annotation, spectral and taxonomy data for chemical reports.

The loss of experimental and semantic (ontological) information using traditional analogue publishing methods is tremendous and underestimated. This is especially the case for high resolution mass spectra and NMR spectra [75]. It has been shown that an enforcement for the publication of metadata is possible as in the crystallographic community and the Cambridge Structural Database [76]. Successful fully automated aggregation of crystal structures from open access supplement data has been shown by the CrystalEye project [48]. Data driven research approaches which require access to large amounts of experimental data are not possible with current database collections that are not linked with global and open identifiers and have limited access strategies.

Chemical database providers should allow batch download of compounds or batch matching of large compound data sets, provided that there are no financial or legal restrictions. Chemical and biological database providers should also enhance their database records with PubChem substance or compound IDs and with InChI codes and the hashed InChIKeys to promote a universal connection between modern life sciences and chemistry databases. The problem of differentiation between xenobiotic molecules and metabolites that are produced by the biochemical machinery can only be solved by additional database annotations and use of ontologies. Especially the differentiation of the endo- and exo-metabolome for superorganism like humans, where many compounds are believed to belong to the human enzyme machinery but are actually excreted by the gut microbiome are only solvable by special experimental setups and extended annotations.

Computational approaches based on orthology mapping seem to be the most intuitive and therefore successful methods to increase coverage on small molecules in databases for all taxonomic species. Such computationally predicted metabolites could be included as putative compounds in ChemSpider/PubChem repositories once these chemical databases are enriched with taxonomy, literature and spectral data. Further improvements could then annotate absolute metabolite concentrations reported for specific organs and cellular compartments to allow cross-database and cross-species queries. Only concerted efforts between scientific societies, funding agencies, database providers and journals could break up traditional ways of metabolic reporting. Nevertheless, even single projects like RSC Project Prospect, CrystalEye, SetupX or ChemSpider can lead into the right direction in a pull-strategy, by leading by example.

### Compound annotations from text

(Name; PubChem CID; InChIKey)

Also see [Dataset S1]

2-acetyl-1-pyrroline; CID 522834; DQBQWWSFRPLIAX-UHFFFAOYAG

Vitamin-A; CID 445354; FPIPGXGPPPQFEQ-OVSJKPMPBW

Beta-carotene; CID 5280489; OENHQHLEOONYIE-JLTXGRSLBT

Bisbynin; CID NA; ICHJNTDKHBXTFN-CMZGOGIXBZ

Trans-luteine; CID 5368396; KBPHJBAIARWVSC-DKLMTRRABK

Cholesterol; CID 5997; HVYWMOMLDIMFJA-DPAQBDIFBB

Malathion; CID 4004; JXSJBGJIGXNWCI-UHFFFAOYAK

Chlorpyrifos; CID 2730; SBPBAQFWLVIOKP-UHFFFAOYAG

Ribosylnicotinamide; CID 439924; JLEBZPBDRKPWTD-ARWKKGFBBE

Omeprazol; CID 4594; SUBDBMMJDZJVOS-UHFFFAOYAZ

Rhodopinal; CID 20055178; GOJQFVQXKNNAAY-XQHLYSSHBM

Tegafur; CID 5386; WFWLQNSHRPWKFK-UHFFFAOYAE

Arginine; CID 232; ODKSFYDXXFIFQN-UHFFFAOYAT

## Supporting Information

Dataset S1 Supporting ZIP file for publication. Contains chemical compound structures and figures and other information.
(0.55 MB ZIP)

## References

[1] International Rice Research Institute (IRRI); [http://www.irri.org/]

[2] Kind T, Fiehn O (2008). Hardware and Software Challenges for the Near Future: Structure Elucidation Concepts via Hyphenated Chromatographic Techniques.. Lc Gc N Am.

[3] Kind T, Fiehn O (2006). Metabolomic database annotations via query of elemental compositions: Mass accuracy is insufficient even at less than 1 ppm.. BMC Bioinformatics.

[4] Kind T, Fiehn O (2007). Seven Golden Rules for heuristic filtering of molecular formulas obtained by accurate mass spectrometry.. BMC Bioinformatics.

[5] Hill DW, Kertesz TM, Fontaine D, Friedman R, Grant DF (2008). Mass Spectral Metabonomics beyond Elemental Formula: Chemical Database Querying by Matching Experimental with Computational Fragmentation Spectra.. Analytical Chemistry.

[6] Schymanski EL, Meinert C, Meringer M, Brack W (2008). The use of MS classifiers and structure generation to assist in the identification of unknowns in effect-directed analysis.. Analytica chimica acta.

[7] Chemical Abstracts Database - CAS SciFinder academic program; [http://www.cas.org/]

[8] Dictionary of Natural Products; [http://dnp.chemnetbase.com/]

[9] NCBI Entrez - the Life Sciences Search Engine; [http://www.ncbi.nlm.nih.gov/Entrez/]

[10] PubMed Central Digital Archive; [http://www.pubmedcentral.nih.gov/]

[11] CrossFire Beilstein database; [http://www.beilstein.com/]

[12] Dr. Duke's Phytochemical and Ethnobotanical Database; [http://www.ars-grin.gov/duke/]

[13] SetupX - Study design database for metabolomic projects; [http://fiehnlab.ucdavis.edu:8080/m1/] 17990490

[14] Fiehn O, Wohlgemuth G, Scholz M (2005). Setup and Annotation of Metabolomic Experiments by Integrating Biological and Mass Spectrometric Metadata.. LECTURE NOTES IN COMPUTER SCIENCE.

[15] RiceCyc - Rice Metabolic Pathways; [http://www.gramene.org/pathway/ricecyc.html]

[16] Jaiswal P, Ni J, Yap I, Ware D, Spooner W (2006). Gramene: a bird's eye view of cereal genomes.. Nucl Acids Res.

[17] KEGG Database - Kyoto Encyclopedia of Genes and Genomes; [http://www.genome.jp/kegg/]

[18] Oikawa A, Nakamura Y, Ogura T, Kimura A, Suzuki H (2006). Clarification of Pathway-Specific Inhibition by Fourier Transform Ion Cyclotron Resonance/Mass Spectrometry-Based Metabolic Phenotyping Studies.. Plant Physiol.

[19] KNApSAcK: A Comprehensive Species-Metabolite Relationship Database; [http://kanaya.aist-nara.ac.jp/KNApSAcK/]

[20] Reactome - a curated knowledgebase of biological pathways; [http://www.reactome.org/] 10.1111/j.1538-7836.2012.04930.x PMC3578965 22985186

[21] IBM Chemical Search Engine alpha; [http://chemsearch.almaden.ibm.com/]

[22] SureChem chemical patent search database; [http://www.surechem.org/]

[23] Austin R (2001).

[24] Kind T, Scholz M, Fiehn O http://fiehnlab.ucdavis.edu/projects/Rice_metabolome/.

[25] De Silva LB, Herath WHMW, Gunawardena DSS, Wijesundera RLC, Medis SA (1995). Bisbynin, a novel secondary metabolite from the fungus Stachybotrys bisbyi (Srinivasan) Barron.. Tetrahedron Letters.

[26] Zhou Y, Zhou B, Chen K, Yan SF, King FJ (2007). Large-Scale Annotation of Small-Molecule Libraries Using Public Databases.. Journal of Chemical Information and Modeling.

[27] Wheeler DL, Barrett T, Benson DA, Bryant SH, Canese K (2007). Database resources of the National Center for Biotechnology Information.. Nucl Acids Res.

[28] Williams AJ (2008). Internet-based tools for communication and collaboration in chemistry.. Drug Discovery Today.

[29] NCBI Taxonomy Homepage; [http://www.ncbi.nlm.nih.gov/Taxonomy/]

[30] Plant Ontology Consortium; [http://www.plantontology.org/]

[31] Edinburgh Human Developmental Anatomy Ontology; [http://genex.hgu.mrc.ac.uk/Databases/HumanAnatomy/]

[32] Fiehn O (2007). Cellular Metabolomics: The Quest for Pathway Structure..

[33] SMILES - Simplified molecular input line entry specification; [http://en.wikipedia.org/wiki/Simplified_molecular_input_line_entry_specification]

[34] Stein SE, Heller SR, Tchekhovski D http://www.iupac.org/inchi/.

[35] LIPID MAPS – LIPID Metabolites And Pathways Strategy; [http://www.lipidmaps.org/]

[36] KEGG BRITE Database; [http://www.genome.jp/kegg/brite.html]

[37] Instant JChem - for structure database management, search and prediction, Instant JChem 2.4, 2008, ChemAxon [http://www.chemaxon.com]

[38] Rhodes J, Boyer S, Kreulen J, Chen Y, Ordonez P (2007).

[39] Caspi R, Foerster H, Fulcher CA, Kaipa P, Krummenacker M (2008). The MetaCyc Database of metabolic pathways and enzymes and the BioCyc collection of Pathway/Genome Databases.. Nucleic Acids Research.

[40] Oryzabase - Integrated Rice Science Database; [http://www.shigen.nig.ac.jp/rice/oryzabase/]

[41] MetaCrop - a detailed database of crop plant metabolism; [http://metacrop.ipk-gatersleben.de/] 10.1093/nar/gkm835 PMC2238923 17933764

[42] Cui Q, Lewis IA, Hegeman AD, Anderson ME, Li J (2008). Metabolite identification via the Madison Metabolomics Consortium Database.. Nat Biotech.

[43] Wishart DS, Tzur D, Knox C, Eisner R, Guo AC (2007). HMDB: the Human Metabolome Database.. Nucl Acids Res.

[44] Smith CA, O'Maille G, Want EJ, Qin C, Trauger SA (2005). METLIN: A Metabolite Mass Spectral Database.. Therapeutic Drug Monitoring.

[45] ChEBI - Chemical Entities of Biological Interest; [http://www.ebi.ac.uk/chebi/]

[46] Ott MA, Vriend G (2006). Correcting ligands, metabolites, and pathways.. BMC Bioinformatics.

[47] Paley SM, Karp PD (2006). The Pathway Tools cellular overview diagram and Omics Viewer.. Nucleic Acids Research.

[48] Arita M (2003). In Silico Atomic Tracing by Substrate-Product Relationships in Escherichia coli Intermediary Metabolism.. Genome Research.

[49] Mu F, Williams RF, Unkefer CJ, Unkefer PJ, Faeder JR (2007). Carbon-fate maps for metabolic reactions.. Bioinformatics.

[50] Heller SR, Stein SE, Tchekhovskoi DV (2005). InChI: Open access/open source and the IUPAC international chemical identifier; 2005.. Amer Chemical Soc.

[51] International Chemical Identifier; [http://en.wikipedia.org/wiki/International_Chemical_Identifier]

[52] InChI Resolver service at Chemspider for the generation and lookup of InChI codes and InChIKeys; [http://inchis.chemspider.com/]

[53] Kidd R (2009). Changing the face of scientific publishing.. Integrative Biology.

[54] Heller SR, McNaught AD http://www.iupac.org/publications/ci/2009/3101/2_heller.html.

[55] Sumner LW, Amberg A, Barrett D, Beale MH, Beger R (2007). Proposed minimum reporting standards for chemical analysis.. Metabolomics.

[56] MIBBI: Minimum Information for Biological and Biomedical Investigations; [http://www.mibbi.org/]

[57] Taylor CF, Field D, Sansone SA, Aerts J, Apweiler R (2008). Promoting coherent minimum reporting guidelines for biological and biomedical investigations: the MIBBI project.. Nature Biotechnology.

[58] Guha R, Howard MT, Hutchison GR, Murray-Rust P, Rzepa H (2006). Blue Obelisk - Interoperability in chemical informatics.. Journal of Chemical Information and Modeling.

[59] Murray-Rust P (2008). Chemistry for everyone.. Nature.

[60] Strasser BJ (2008). GENETICS: GenBank–Natural History in the 21st Century?. Science.

[61] Baker DB, Horiszny JW, Metanomski WV (1980). History of Abstracting at Chemical Abstracts Service.. Journal of Chemical Information and Computer Sciences.

[62] Flaxbart D http://www.istl.org/07-winter/viewpoints.html.

[63] Casher O, Rzepa HS (2006). SemanticEye: A Semantic Web Application to Rationalize and Enhance Chemical Electronic Publishing.. Journal of Chemical Information and Modeling.

[64] Apodaca R http://depth-first.com/articles/tag/metadata.

[65] Chem4Word - semantically rich chemistry information in Word 2007 documents; [http://research.microsoft.com/en-us/projects/chem4word/]

[66] Corbett P, Murray-Rust P, Berthold MR, Glen R, Fischer I (2006). High-throughput identification of chemistry in life science texts..

[67] Willighagen EL, O'Boyle NM, Gopalakrishnan H, Jiao D, Guha R (2007). Userscripts for the life sciences.. Bmc Bioinformatics.

[68] Filippov IV, Nicklaus MC (2009). Optical Structure Recognition Software To Recover Chemical Information: OSRA, An Open Source Solution.. Journal of Chemical Information and Modeling.

[69] McDaniel JR, Balmuth JR (1992). KEKULE - OCR OPTICAL CHEMICAL (STRUCTURE) RECOGNITION.. Journal of Chemical Information and Computer Sciences.

[70] Ibison P, Jacquot M, Kam F, Neville AG, Simpson RW (1993). CHEMICAL LITERATURE DATA EXTRACTION - THE CLIDE PROJECT.. Journal of Chemical Information and Computer Sciences.

[71] Park J, Rosania G, Shedden K, Nguyen M, Lyu N (2009). Automated extraction of chemical structure information from digital raster images.. Chemistry Central Journal.

[72] Kuhn S, Helmus T, Lancashire RJ, Murray-Rust P, Rzepa HS (2007). Chemical Markup, XML, and the World Wide Web. 7. CMLSpect, an XML Vocabulary for Spectral Data.. Journal of Chemical Information and Modeling.

[73] Downing J, Murray-Rust P, Tonge AP, Morgan P, Rzepa HS (2008). SPECTRa: The Deposition and Validation of Primary Chemistry Research Data in Digital Repositories.. Journal of Chemical Information and Modeling.

[74] RSC Project Prospect; [http://www.rsc.org/Publishing/Journals/ProjectProspect/]

[75] Steinbeck C, Kuhn S (2004). NMRShiftDB - compound identification and structure elucidation support through a free community-built web database.. Phytochemistry.

[76] Allen FH (2002). The Cambridge Structural Database: a quarter of a million crystal structures and rising.. Acta Crystallogr Sect B-Struct Sci.

