# Supplementary figures and images for: How Large Is the Metabolome? A Critical Analysis of Data Exchange Practices in Chemistry
Source: PLoS One. 2009 May 5;4(5):e5440. doi: 10.1371/journal.pone.0005440 (PMC2673031; doi:10.1371/journal.pone.0005440)

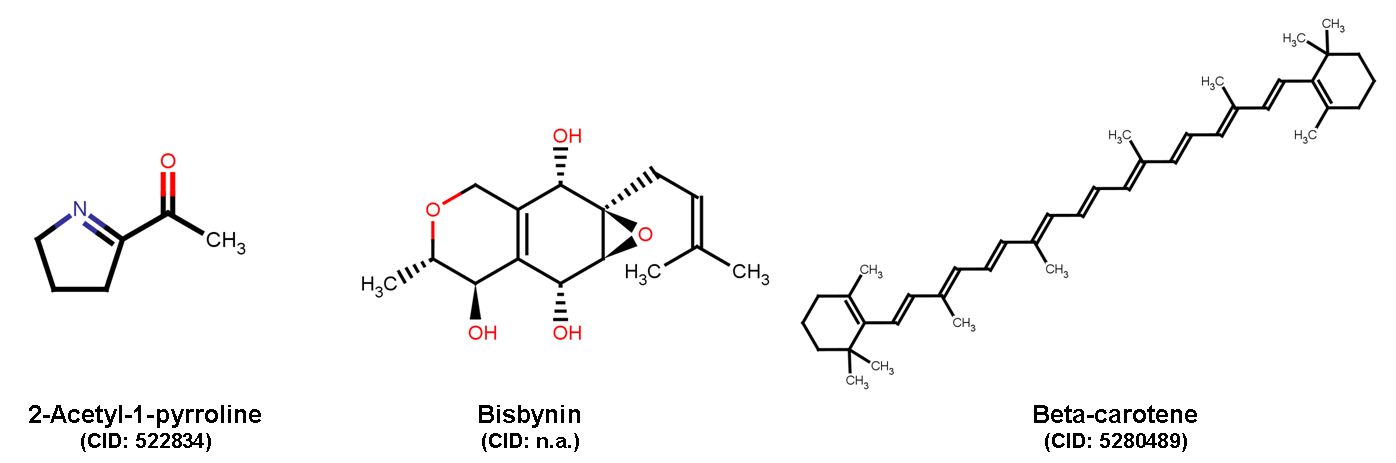

Supplement: Dataset S1 — Supporting ZIP file for publication. Contains chemical compound structures and figures and other information. (0.55 MB ZIP) [file pone.0005440.s001.zip › Supplement-PLOS/Figure1-rice-300dpi.tif]

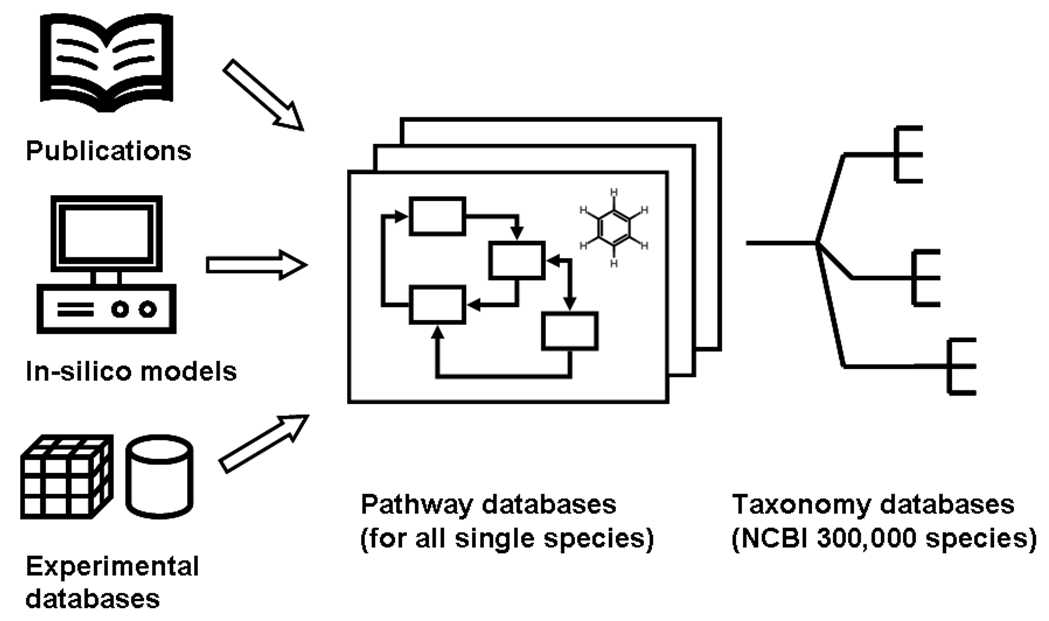

Supplement: Dataset S1 — Supporting ZIP file for publication. Contains chemical compound structures and figures and other information. (0.55 MB ZIP) [file pone.0005440.s001.zip › Supplement-PLOS/Figure2-rice-300dpi.tif]

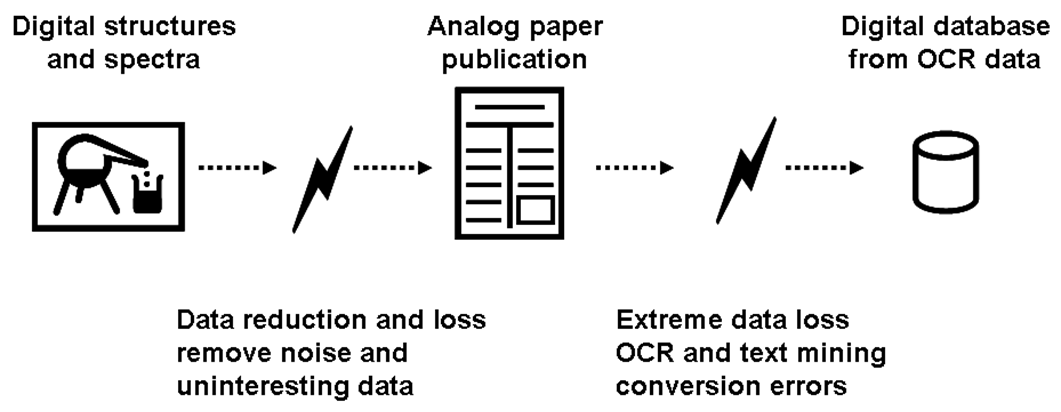

Supplement: Dataset S1 — Supporting ZIP file for publication. Contains chemical compound structures and figures and other information. (0.55 MB ZIP) [file pone.0005440.s001.zip › Supplement-PLOS/Figure3-rice-300dpi.tif]
